# SARS-CoV-2 Inactivation Simulation Using 14 MeV Neutron Irradiation

**DOI:** 10.3390/life11121372

**Published:** 2021-12-09

**Authors:** Fang Liu, Zhengtong Zhong, Bin Liu, Tianze Jiang, Hongchi Zhou, Guanda Li, Xin Yuan, Peiguang Yan, Fenglei Niu, Xiaoping Ouyang

**Affiliations:** 1 Beijing Key Laboratory of Passive Safety Technology for Nuclear Energy, School of Nuclear Science and Engineering, North China Electric Power University, Beijing 102206, China; liuf@ncepu.edu.cn (F.L.); zhongzt@ncepu.edu.cn (Z.Z.); Jiangtz@ncepu.edu.cn (T.J.); zhouhongchi@ncepu.edu.cn (H.Z.); liguanda@ncepu.edu.cn (G.L.); yuanxin@ncepu.edu.cn (X.Y.); niufenglei@ncepu.edu.cn (F.N.); 2 Shenzhen Key Laboratory of Laser Engineering, College of Physics and Optoelectronic Engineering, Shenzhen University, Shenzhen 518060, China; yanpg@szu.edu.cn; 3 Radiation Detection Research Center, Northwest Institute of Nuclear Technology, Xi’an 710024, China; oyxp2003@aliyun.com; 4 Department of Engineering Physics, Tsinghua University, Beijing 100084, China

**Keywords:** SARS-CoV-2 inactivation, COVID-19, Monte Carlo simulation, neutron

## Abstract

The SARS-CoV-2 virus is deadly, contagious, can cause COVID-19 disease, and endangers public health and safety. The development of SARS-CoV-2 inactivation technology is crucial and imminent in current pandemic period. Neutron radiation is usually used to sterilize viruses because neutron radiation is 10 times more effective than gamma-rays in inactivating viruses. In this work we established a closed SARS-CoV-2 inactivation container model by the Monte Carlo method and simulated the inactivation performance by using several different neutrons sources. To study the effects of inactivation container factors, including the reflector thickness, the type of the reflector material, the SARS-CoV-2 layer area and the distance from the radiation source on the energy deposition of a single neutron particle in SARS-CoV-2 sample, we simulated the neutron energy deposition on a SARS-CoV-2 sample. The simulation results indicate that the saturated thicknesses of reflector materials for graphite, water and paraffin are approximately 30 cm, 15 cm, and 10 cm, respectively, and the energy deposition (radiation dose) becomes larger when the SARS-CoV-2 layer area is smaller and the SARS-CoV-2 layer is placed closer to the neutron source. The calculated single-neutron energy deposition on 10 × 10 cm^2^ SARS-CoV-2 layer is about 3.0059 × 10^−4^ MeV/g with graphite as the reflection layer, when the 14 MeV neutron source intensity is 10^12^ n/s and the SARS-CoV-2 layer is 5 cm away from the neutron source. If the lethal dose of SARS-CoV-2 is assumed as the IAEA recommended reference dose, 25 kGy, the SARS-CoV-2 could be decontaminated in about 87 min, and the sterilization time could be less than 52 s if the 14 MeV neutron intensity is increased to 10^14^ n/s.

## 1. Introduction

**Figure 1:**
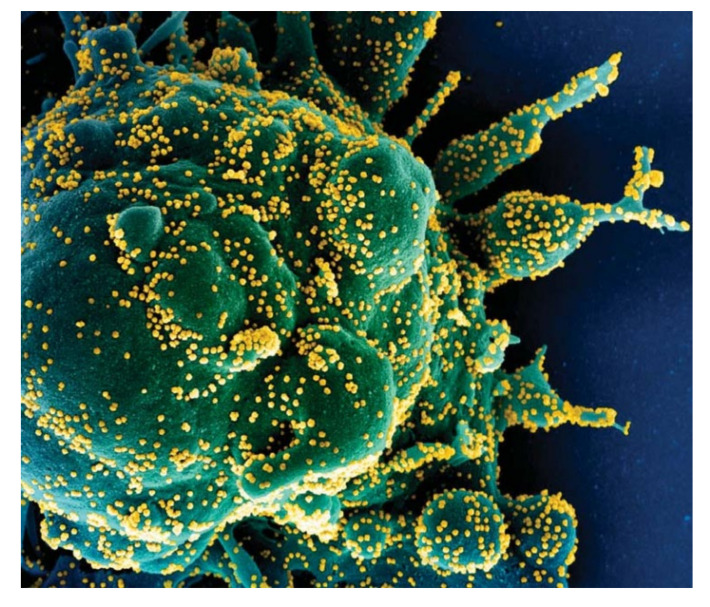
Scanning electron micrograph of a cell (green) heavily infected with SARS-CoV-2 virus particles (yellow) [1].

The severe acute respiratory syndrome coronavirus 2 (SARS-CoV-2) [1] is currently sweeping round the world and has caused the coronavirus disease 2019 (COVID-19) pneumonia (shown in Figure 1 [1]). This is a new strain of coronavirus, which is contagious, can spread rapidly and widely through carriers, and has threatened human lives globally.

Although the coronavirus was discovered in the 1930s [2], coronaviruses gained worldwide attention when the severe acute respiratory syndrome outbreak shook the world in 2003. Biologists’ interest in this family of viruses grew in the aftermath of the epidemic of 2003, leading to the identification of many new coronavirus family members. The coronavirus break out in 2003 also gave a hint of the ability of coronaviruses to jump across species. Before gaining worldwide attention for threatening public health in 2003, the diseases associated with coronaviruses were mainly of veterinary interest. Coronaviruses can infect a wide variety of creatures, such as mammals and birds [3], causing respiratory and enteric diseases and, in some rarer cases, hepatitis and neurologic disease. Infection can be acute or persistent.

Coronaviruses are enveloped, spherical or pleiomorphic viruses, with a radius of approximately 60 nm. The most distinctive feature of coronaviruses is the club-shaped spike projections emanating from the surface of the virion. These spikes are a defining feature of the virion and give them the appearance of a corona, giving the name, coronaviruses. Within the shell of the virion is the nucleocapsid. Coronaviruses have helically symmetrical nucleocapsids, which is uncommon among positive-sense RNA viruses, but far more common in negative-sense RNA viruses. The coronavirus in the current pandemic can spread from an infected individual’s mouth or nose in small liquid particles when they cough, sneeze, speak, sing or breathe in closed or open areas. These particles range from larger respiratory droplets to smaller aerosols. It is important to practice respiratory etiquette, for example by coughing into a flexed elbow, and if an infected person feels unwell, to stay home and self-isolate until they recover [4,5].

Most people infected with the coronavirus will experience mild to moderate respiratory illness and recover without requiring special medical treatment. However, some will become seriously ill and require medical attention. Older people and those with underlying medical conditions like cardiovascular disease, diabetes, chronic respiratory disease, or cancer are more likely to develop serious illness. However, any person, at any age, can get sick with coronavirus and become seriously ill or die. The coronavirus pandemic around world has caused millions of deaths as well as lasting health problems in some individuals who have survived the illness. Although COVID-19 vaccines have been authorized for emergency use by the most countries and vaccination programs are in progress across the many parts of the world, the development of SARS-CoV-2 inactivation technology is crucial and imminent in current pandemic period. Effective vaccines against SARS-CoV-2 in humans are still in research and development and some of the promising vaccines have been pre-approved and used in some countries [6,7]. When bulk goods, such as luggage, medical instrumentations, tools, etc., are contaminated by SARS-CoV-2 in public areas, the traditional medical procedures, including pasteurization, alcohol and ultraviolet irradiation, which are generally used to kill common pathogenic bacteria [8], just do not work. Using alcohol to sterilize SARS-CoV-2 contamination is only feasible in some cases of surface contamination; it does not work effectively against SARS-CoV-2 hidden inside sealed containers, luggage, food, and so on. 

Compared with traditional sterilization methods, radiation sterilization technology [9] has the advantages of short time, high efficiency, no damage, low energy consumption, and suitability for large-scale sterilization. There are chemical and physical techniques for virus inactivation and radiation disinfection or sterilization is a physical method, acting mainly through the inactivation of viruses by gamma rays and electron beam irradiation [10]. There are many studies using irradiation technologies such as X-rays, neutron irradiation, etc., to inactivate different viruses [11,12,13].

The γ-ray irradiation technique has been used to kill bacteria and anthrax hidden in sealed metal equipment or large luggage because the γ-ray irradiation is more penetrative than electron beams [10]. However, theoretical study showed that the neutron irradiation yielded higher sterilization efficiency for anthrax spores than γ-ray irradiation. Neutrons can penetrate sealed equipment to kill both anthrax spores, not only on surfaces, but also those hidden inside bulk goods or luggage, because neutrons have no charge and can penetrate. Simulation study has shown that 2.5 MeV neutron irradiation from a D-D neutron generator can sterilize all anthrax spores in a sample within approximately 1 min [14]. 

The radiation particle type and energy affect the irradiation inactivation efficiency significantly because they have quite different weighting factors. The radiation weighting factor represents the relative radiation damage to the tissue or organ resulting from the unit deposition energy. The radiation damage depends not only on the deposition energy, but also on the radiation type and energy. The radiation weighting factor of photon and electron particles is 1, which is independent of the energy of the radiation. However, for neutron radiation, the weighting factor is energy-dependent, and its value may be from 5 to 20. The weighting factor of 14 MeV neutrons is 10, compared with electrons and γ-rays, the weighting factor of which is 1, meaning that the neutrons can cause 10 times the damage to organisms that γ-rays can for the same energy deposition or radiation dose. The virus inactivation is mainly caused by damage from the irradiation to the viral nucleic acid, including RNA and DNA. A single-strand break (for single-stranded viruses) or a double-strand break led by irradiation damage is enough to kill the viruses [15].

Several different neutron sources can create enough neutron irradiation to sterilize all kind of viruses. The common neutron sources used to sterilize the virus contamination are radioisotope neutron sources and monoenergetic neutron generators. There are many radioisotope neutron sources, the typical radioisotope neutrons sources used in nuclear technology are ^252^Cf (Californium-252 neutron source) and ^241^Am-Be (Americium beryllium neutron source). The half-life of ^252^Cf neutron source is about 2.6 years, while the half-life of ^241^Am-Be is 432.2 years. The neutron energy spectrum emitted from ^252^Cf neutron source is similar to the fission neutron spectrum, with 2 MeV average neutron energy. ^241^Am-Be neutron source emits both neutrons and α particles, with 4.5 MeV average neutron energy. 

Neutron generators are electronic devices that contain compact linear particle accelerators and produce neutrons by fusing isotopes of hydrogen atoms together. The fusion reactions take place in these electronic devices by accelerating either deuterium, tritium, or a mixture of these two isotopes into a metal hydride target which also contains deuterium, tritium, or a mixture of these isotopes. There are two different types of monoenergetic neutron generators, D-D neutron generators and T–D neutron generators [16]. Fusion of deuterium atoms (D + D) results in the formation of a He-3 ion and a neutron with a kinetic energy of approximately 2.5 MeV. Fusion of a deuterium and a tritium atom (D + T) results in the formation of a He-4 ion and a neutron with a kinetic energy of approximately 14.1 MeV [17]. Neutron generators have wide applications in physics, nuclear technology, materials analysis, and medicine.

The use of neutron irradiation in the treatment of food and food packaging is regulated by the U.S. Food & Drug Administration (FDA), which controls the energy of neutrons used for inspection of food and packaged food ranging from 1 MeV to 14 MeV [18]. In the coronavirus inactivation simulation calculations, we choose a 14 MeV neutron generator to sterilize SARS-CoV-2, because it has high neutron yield and monochromatic energy spectrum and can generate pulsed neutrons. The 14 MeV neutron generator can be turned off to stop neutron generation, so it is easy to shield, store and transport. We set up a SARS-CoV-2 inactivation model and placed the neutron source above the SARS-CoV-2 layer at a height of 5 cm. In MCNP simulation calculations, we consider the effects of the simulation set-up components, including the reflector material, the reflector thickness, the SARS-CoV-2 layer area, and the distance between the samples and the neutron source [16].

In this study, we set up a simulation model by MCNP code [19] and calculated the single-neutron energy deposition, the effect of the reflector materials and reflector thickness, and the incident neutron energy that is needed to sterilize SARS-CoV-2 contamination. The simulation results present the effect of reflector materials, reflector thickness on the neutron energy deposition in SARS-CoV-2 layer and illustrate the available estimated sterilization time using 14 MeV neutron generator. 

## 2. Materials and Methods

The SARS-CoV-2 virion is composed of 29 proteins in total including 16 non-structural proteins and nine accessory proteins [20], which are hydrogen (proton)-rich materials. Neutrons and hydrogen atoms have high nuclear interaction cross sections, which indicates the possibility of interaction between neutrons and hydrogen atoms is high and neutrons can cause damage to SARS-CoV-2 DNA strands. SARS-CoV-2 belongs to the coronavirus family that includes the other well-known viruses SARS-CoV and MERS-CoV [21], and is an enveloped, single-stranded, positive-sense RNA virus of similar size to other coronaviruses [20]. 

RNA has a single chain structure that is easy to break and recombine. When neutrons hit the SARS-CoV-2 virus, they will interact with the nuclei of the hydrogen, oxygen and carbon atoms within the proteins of the SARS-CoV-2 virion. The neutrons tend to knock protons out of hydrogen nuclei meanwhile and transfer their energy to the protons. The scattered protons are ionizing radiation and interact with biological molecules as they go through the SARS-CoV-2 layer. In this way, the protons deposit their energy to biological molecules along their tracks by ionizing or exciting surrounding viral molecules, and the deposited energy induces the biological damage. The damage severity is directly related to the local rate of energy deposition along the protons penetration track [17]. The biological damage can cause the single chain of RNA to break. The viruses lack enzymes and are therefore unable to repair any damage in their RNA and DNA [22]. This is the reason why we decide to use penetrating neutron radiation to sterilize SARS-CoV-2 contamination.

**Figure 2:**
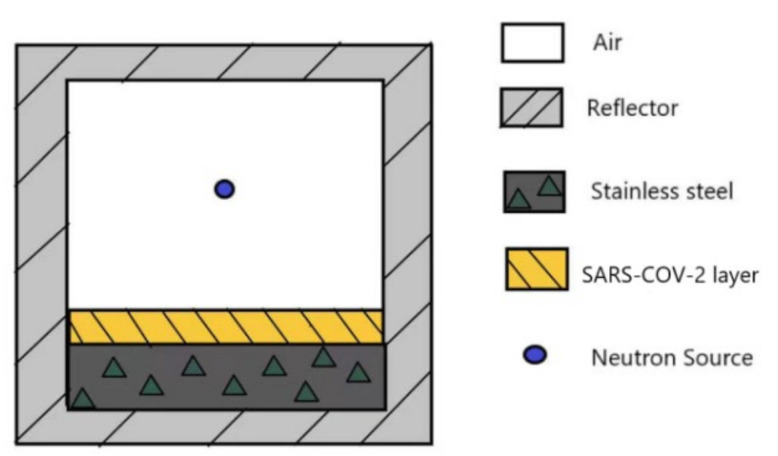
The SARS-CoV-2 inactivation simulation model.

We set up a virus inactivation model shown in Figure 2 to simulate inactivation of SARS-CoV-2 using 14 MeV neutrons by MCNP code. This model consists of a stainless-steel container, a surrounding reflector layer, a SARS-CoV-2 layer, and 14 MeV neutron sources. We used water, paraffin, and graphite as the reflector materials because these three materials are hydrogen-rich and good for reflecting and “slowing down” neutrons.

Figure 2 shows the simulation model of neutron irradiation inactivation of SARS-CoV-2. In this model, the SARS-CoV-2 samples are supported by a stainless-steel frame at the bottom of the container; The SARS-CoV-2 layer area is 10 × 10 cm^2^ and the thickness of the SARS-CoV-2 layer is 1 mm. We used a 14 MeV neutron source in the simulation model.

For the simulation calculations, we set up a simulation model in MCNP code to calculate the average energy deposition in the SARS-CoV-2 layer. For the simulation calculations, we wrote the input file in MCNP code to calculate the single-neutron energy deposition in the SARS-CoV-2 sample. The neutrons interact with the SARS-CoV-2 sample and deposit their energy within the SARS-CoV-2 sample mainly through neutron–proton collisions. We calculated the single-neutron energy deposition in the SARS-CoV-2 layer with different reflector thicknesses, various reflector materials, and different distances between the SARS-CoV-2 sample and the neutron source. In the simulation, we ignored the energy deposition of γ-ray irradiation which is created by the neutrons’ interaction with the reflector materials. 

## 3. Results and Discussions

**Figure 3:**
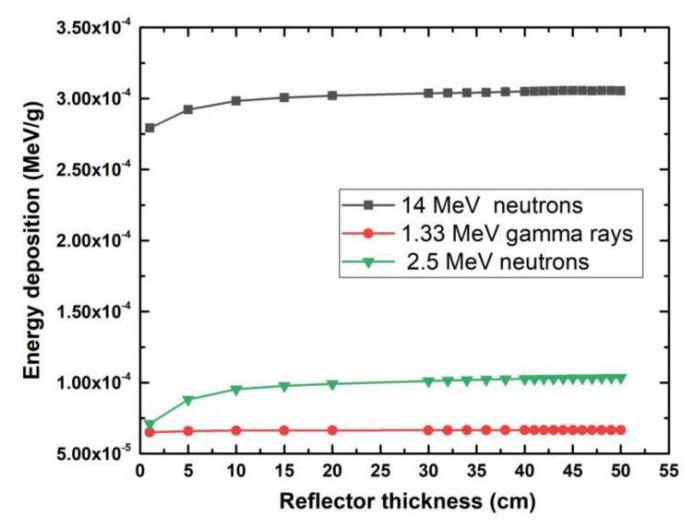
Single-energy deposition in the SARS-CoV-2 layer using three irradiation sources with graphite as the reflector.

We calculated the average energy deposition per particle in the SARS-CoV-2 sample using 14 MeV and 2.5 MeV neutrons, and 1.33 MeV gamma rays with the source 5 cm away from the SARS-CoV-2 sample, as shown in Figure 3. The calculation results show that the 14 MeV neutrons deposit more energy than the other two irradiation sources, therefore we choose 14 MeV neutrons to irradiate SARS-CoV-2 sample in the simulation model.

**Figure 4:**
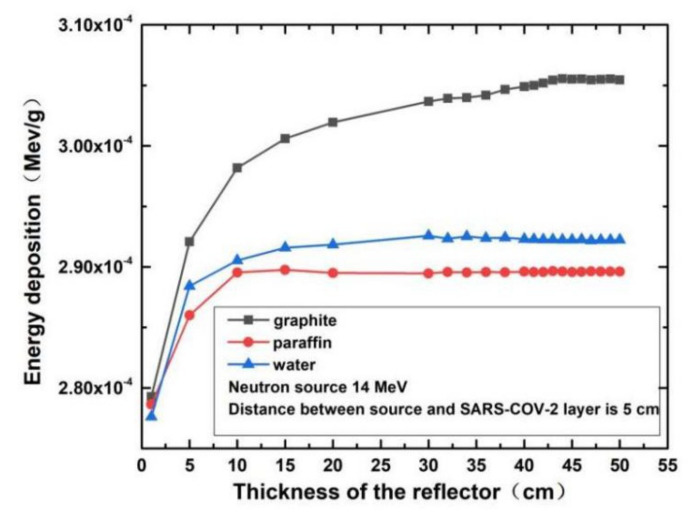
The single-energy deposition on SARS-CoV-2 layer for three reflecting materials.

**Figure 5:**
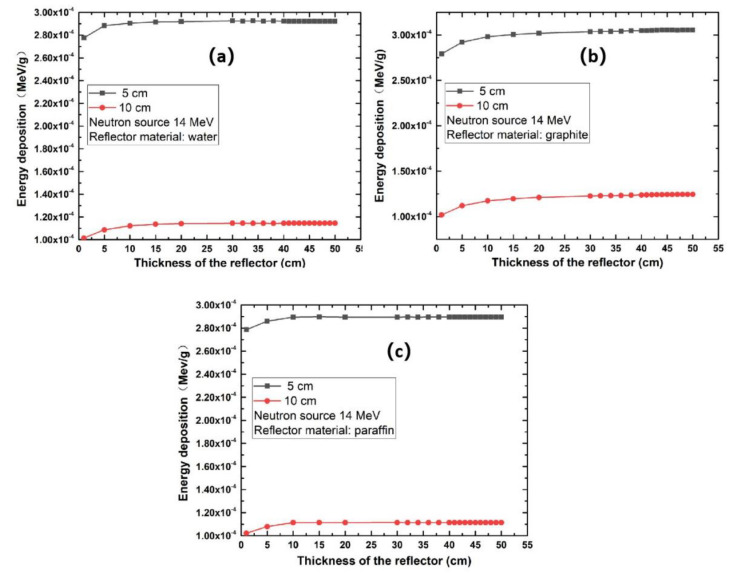
The single-energy deposition on SARS-CoV-2 layer using (**a**) water, (**b**) graphite, and (**c**) paraffin reflector.

Figure 4 shows that the reflector materials had an effect on the neutron energy deposition. Figure 5a–c show the effect of the distance between the neutron source and the SARS-CoV-2 sample on the neutron energy deposition with water, graphite, and paraffin as reflectors, respectively.

### 3.1. The Effect of the Reflector Materials and Reflector Thickness on the Neutron Energy Deposition in the SARS-CoV-2 Layer

The simulation results of energy deposition using 14 MeV neutrons for three reflector materials are shown in Figure 4. We can see that the single-neutron energy deposition increases rapidly when the reflector thickness is less than 10 cm for the three materials used (water, paraffin or graphite). However, the single-neutron energy deposition shows almost no further increase when the thickness of reflector materials reaches a certain value; we call this reflector thickness the saturated thickness. Therefore, using reflector materials thicker than the saturated thickness to increase the single-neutron energy deposition is ineffective.

The results in Figure 4 show that the saturated thickness varies with different reflector materials. The saturated thicknesses of graphite, water, and paraffin are approximately 30 cm, 15 cm, and 10 cm, respectively. In the anthrax sterilization research using 2.5 MeV neutrons radiation, the saturated thickness was about 15 cm [17]. The saturated thickness is not closely related to the distance between the neutron source and the SARS-CoV-2 sample. From Figure 5a–c, we can see that the saturated thicknesses for graphite, water, and paraffin reflecting materials at 5 cm and 10 cm distances are still about 30 cm, 15 cm, and 10 cm, respectively, which shows that the saturated thickness is independent of the distance between the neutron source and the SARS-CoV-2 sample. This independence phenomenon also appeared in the anthrax sterilization work. 

### 3.2. The Effect of the Distance between the Neutron Source and SARS-CoV-2 Sample on the Neutron Energy Deposition 

We can see from the results shown in Figure 5a–c that the single-neutron energy deposition in the SARS-CoV-2 sample becomes greater when the SARS-CoV-2 layer is set much closer to the neutron source. At the same time, we find that these distance-related results are somewhat off the inverse-square law. However, the result is still reasonable because the SARS-CoV-2 sample was in a square shape, meaning each protein molecule had different distance to the neutron source, and we also used the reflector in the model. Both these factors can cause the neutron energy deposition to be off the inverse-square law somewhat. In real neutron-based inactivation of SARS-CoV-2 experimental set-up, it would be necessary to place the neutron source as close as possible to the SARS-CoV-2 area and to use a reflector to increase the energy deposition in the SARS-CoV-2 layer.

### 3.3. The Effect of the SARS-CoV-2 Layer Area on the Neutron Energy Deposition

**Figure 6:**
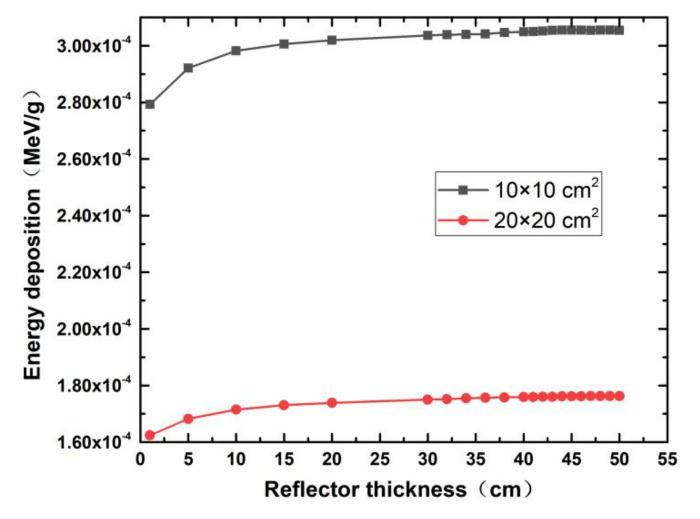
The single-energy deposition on SARS-CoV-2 samples with different layer areas using the graphite reflector.

Figure 6 illustrates the energy deposition on SARS-CoV-2 samples with different layer areas using 14 MeV neutrons and the graphite reflector. It is clear that the energy deposition in the SARS-CoV-2 sample with an area of 20 × 20 cm^2^ is less than that of the 10 × 10 cm^2^ SARS-CoV-2 layer. This is because the average distance between the protein molecules in the SARS-CoV-2 layer and the neutron source becomes larger with the increase in the SARS-CoV-2 layer area, and the lower average neutron penetration into the SARS-CoV-2 layer leads to lower energy deposition. Therefore, we suggest that medical products contaminated by SARS-CoV-2 should be placed around the neutron source in a circle to improve the sterilization efficiency.

### 3.4. The Sterilization Time Estimation of SARS-CoV-2 Using Neutron Irradiation 

The virus damage induced by ionizing radiation is due to chemical alteration of the biological molecules, by the ionization or excitation caused by their interaction with the radiation. In the ionization or excitation process, the ionizing radiation particles deposit their energy onto the biological molecules. When a neutron collides with a proton in SARS-CoV-2, it transfers all its energy to the proton because a neutron has the same mass as a hydrogen nucleus (proton), thus it deposits all its energy in the SARS-CoV-2 virion. Therefore, compared with other forms of radiation, neutrons can create greater biological damage to SARS-CoV-2. Neutrons also collide with the oxygen and carbon nuclei in SARS-CoV-2. However, the oxygen and carbon nuclei are more than 10 times heavier than a neutron; therefore, during collision only a small part of the neutron’s energy transfers to the oxygen and carbon nuclei. The energy transfer in neutron–proton collisions dominates the energy transfers to SARS-CoV-2 when irradiated by neutrons, so the neutron–proton collision is the dominant biological damage mechanism within SARS-CoV-2.

The inactivation cross-section of viruses relates to the irradiation dose. The typical survival curves of viruses [23] show that the survival rates of C16 bacteriophages [13] and the foot-and-mouth-disease picornavirus (FMDV) [24] reach the in order of 1.0 × 10^−2^ when the irradiation dose is 2 kGy and 10 kGy, respectively. The study of γ-ray irradiation used to disinfect anthrax suggested that a dose of 2.0 × 10^6^ rad was recommended to kill the most anthrax. The SI equivalent of 2.0 × 10^6^ rad is 20 kGy [9]. 

The International Atomic Energy Agency (IAEA) has suggested that when the pollution level and types of contaminated microorganisms cannot be confirmed, the standard irradiation dose can be set to 25 kGy [25]. The choice of 25 kGy for sterilization using gamma radiation was first suggested in 1959 by Artandli. The dose was proposed based on minimum killing dose of medical products for about 150 microbial species. 25 kGy was selected as the dose for sterilization because it is 40% above the minimum dose required to kill the resistant microorganisms [26]. Accordingly, 25 kGy is the minimum irradiation dose established for sterilization. Radiation sterilization at a dose of 25 kGy provides such a high safety factor that testing for sterility is generally considered superfluous. Therefore, it is reasonable to use the reference dose 25 kGy as the lethal dose for SARS-COV-2. When reference dose irradiated by γ rays is 25 kGy, the lethal dose for SARS-COV-2 irradiated by neutrons becomes 2.5 kGy, because the weighting factor of neutrons is 10 and γ rays’ is 1. Thus, the necessary dose using neutrons irradiation to kill SARS-COV-2 virus is 25/10 = 2.5 kGy and the no survival reference dose level D for SARS CoV-2 using neutron irradiation is:

D = 2500 Gy(1)

It can be seen from the simulation data shown in Figure 2, Figure 3, Figure 4, Figure 5 and Figure 6 that the highest damage to the SARS-COV-2 caused by neutrons is with the 14 MeV neutron generator as the radiation source, graphite as the reflection layer (saturated thickness), the SARS-COV-2 layer area of 10 × 10 cm^2^, the distance between neutron source and SARS-CoV-2 layer of 5 cm. Under these conditions, the energy deposition of a single neutron in SARS-COV-2 is about 3.0059 × 10^−4^ MeV/g (see Figure 6 for detail). Using this, we calculated the time needed to kill the SARS-CoV-2.

The estimated sterilization time of the SARS-COV-2 by using 14 MeV neutron irradiation can be calculated as [27]:

(2) $$t=\frac{D(Gy)}{d(\mathrm{MeV}/g)\times 10^{6}\times 1.6\times 10^{-19}\times 10^{3}\times Q\times A(n/s)\times 60}\;(\mathrm{Minutes})$$

where, D is the assumed lethal dose for SARS-COV-2, which is about 2500 Gy (2500 J/kg) provied in Equation (1); Q is the neutron weighting factor, which for 14.0 MeV energy is 10; d is the single-neutron energy deposition in the SARS-COV-2; and A is the intensity of radioactive neutrons.

The intensity of the neutron source A in this study is 10^12^ n/s, and the 14 MeV neutron energy deposition d is about 3.0059 × 10^−4^ MeV/g in Figure 6. We inserted these A and d parameters into Equation (2), and the estimated sterilization time is 87 min. The calculated sterilization time shrinks to about 52 s if the neutron intensity increases to 10^14^ n/s, which is feasible because a D-D neutron generator with 10^14^ n/s neutron intensity has been successfully developed [28].

## 4. Conclusions

In this paper, the effect of reflector materials, reflector thickness and the SARS-CoV-2 layer area on the neutron energy deposition in a SARS-CoV-2 layer are studied. In order to focus on the neutron energy deposition in the SARS-CoV-2 layer when the neutron irradiation is used to sterilize the SARS-CoV-2 and minimize the time of the simulation calculation, the energy deposition in the SARS-CoV-2 by other means, such as gamma rays from the surrounding reflector material was ignored when we used MCNP code to do the simulation calculation.

As can be seen from the simulation data, the greater the thickness of the reflector layer is, the more neutron energy is deposited. The neutron energy deposition in the SARS-CoV-2 sample increases with increasing thickness of the reflector, when the thickness of the reflector reaches the saturated thickness, further increases in the thickness of the reflector have little impact on the neutron energy deposition.

The different reflector materials also have different effects on the neutron energy deposition in the SARS-CoV-2 sample. Among all the three reflector materials, graphite gives the maximum neutron energy deposition in the SARS-CoV-2 sample. The saturated thickness of paraffin is only 33% that of graphite, and water is only 50% of graphite. Considering all the factors that affect the neutron energy deposition in the SARS-CoV-2 sample, graphite has the best reflecting effect to sterilize the SARS-CoV-2 viruses, but it has a high density, and is therefore hard to transport. Although water’s neutron-reflecting effect is not as good as that of the graphite, water is readily available anywhere and anytime, thus water may be the ideal reflector material for sterilization of SARS-CoV-2 contamination.

Our calculation results also show that smaller the area of the SARS-CoV-2 layer is, the greater the average energy deposition. This is because the average distance between the protein molecules in SARS-CoV-2 layer and neutron source becomes greater as the SARS-CoV-2 layer area increases, and fewer neutrons penetrate the SARS-CoV-2 layer, leading to lower average neutron energy deposition in the SARS-CoV-2 sample.

In our simulation calculation, a 14 MeV neutron generator was used as the radiation source, the sample area of the SARS-CoV-2 layer was 10 × 10 cm^2^, and the distance between the neutron source and the SARS-CoV-2 sample was set to 5 cm. The single-neutron energy deposition in the SARS-CoV-2 sample was about 3.0059 × 10^−4^ MeV/g. The lethal dose of the SARS-CoV-2 is 25 kGy. Our calculation results show that SARS-CoV-2 can be completely sterilized by 14 MeV neutron irradiation within about 87 min, and the estimated sterilization time shrinks to about 52 s if the neutron intensity is increased to 10^14^ n/s.

## Data Availability

Available on request.
